# Species-specific plant–soil feedbacks alter herbivore-induced gene expression and defense chemistry in *Plantago lanceolata*

**DOI:** 10.1007/s00442-018-4245-9

**Published:** 2018-08-14

**Authors:** Feng Zhu, Robin Heinen, Martijn van der Sluijs, Ciska Raaijmakers, Arjen Biere, T. Martijn Bezemer

**Affiliations:** 1 Department of Terrestrial Ecology, Netherlands Institute of Ecology (NIOO-KNAW), Droevendaalsesteeg, 6708PB Wageningen, The Netherlands; 2 Institute of Biology, Section Plant Ecology and Phytochemistry, Leiden University, P.O. Box 9505, 2300RA Leiden, The Netherlands

**Keywords:** *Mamestra brassicae*, Soil legacy, Iridoid glycosides, Secondary metabolites, Plant defense

## Abstract

Plants actively interact with antagonists and beneficial organisms occurring in the above- and belowground domains of terrestrial ecosystems. In the past decade, studies have focused on the role of plant–soil feedbacks (PSF) in a broad range of ecological processes. However, PSF and its legacy effects on plant defense traits, such as induction of defense-related genes and production of defensive secondary metabolites, have not received much attention. Here, we study soil legacy effects created by twelve common grassland plant species on the induction of four defense-related genes, involved in jasmonic acid signaling, related to chewing herbivore defense (LOX2, PPO7), and in salicylic acid signaling, related to pathogen defense (PR1 and PR2) in *Plantago lanceolata* in response to aboveground herbivory by *Mamestra brassicae*. We also assessed soil legacy and herbivory effects on the production of terpenoid defense compounds (the iridoid glycosides aucubin and catalpol) in *P. lanceolata*. Our results show that both soil legacy and herbivory influence phenotypes of *P. lanceolata* in terms of induction of *Pl* PPO7 and *Pl* LOX2, whereas the expression of *Pl* PR1 and *Pl* PR2-1 is not affected by soil legacies, nor by herbivory. We also find species-specific soil legacy effects on the production of aucubin. Moreover, *P. lanceolata* accumulates more catalpol when they are grown in soils conditioned by grass species. Our study highlights that PSF can influence aboveground plant–insect interactions through the impacts on plant defense traits and suggests that aboveground plant defense responses can be determined, at least partly, by plant-specific legacy effects induced by belowground organisms.

**Electronic supplementary material:**

The online version of this article (10.1007/s00442-018-4245-9) contains supplementary material, which is available to authorized users.

## Introduction

As plants are members of complex communities, they simultaneously interact with both antagonists and beneficial organisms occurring both above and below the ground (Pieterse et al. 2013; Biere and Goverse 2016). To cope with challenges by harmful pathogens and insect herbivores, plants have evolved a complex immune system that modulates plant defensive responses, from recognition of alien molecules or signals from damaged plant cells to activation of effective immune responses against the attackers (Jones and Dangl 2006; Howe and Jander 2008). The phytohormones jasmonic acid (JA), salicylic acid (SA) and ethylene (ET) act as major players in coordinating the signaling pathways involved in multi-trophic species interactions among plants, microbes, and insects (Anand et al. 2008; Erb et al. 2012; Pieterse et al. 2012). In addition, beneficial relationships between plants and soil microbes are common in nature as well, improving plant growth or enhancing the plant’s ability to cope with biotic or abiotic stress (Pineda et al. 2010; Pieterse et al. 2014). Benefits of the associations with microbes to the plants are often based on the growth-promoting effects of beneficial microbes, as well as on the activation of induced systemic resistance (ISR) resulting in sensitization of the plant immune system (priming) for a more efficient activation of plant defenses upon a future attack (Zamioudis and Pieterse 2012). Beneficial rhizosphere microbes can prime the plant for enhanced defense against a broad range of insect herbivores (Van Oosten et al. 2008; Van Wees et al. 2008; Jung et al. 2012; van de Mortel et al. 2012; Pangesti et al. 2013).

The fitness and performance of a plant can depend greatly on the conditions of the soil it grows in (Bardgett and Wardle 2010). The soil is where plants get their water and nutrients from, but it is also the center stage for interactions with a wide range of soil biota. Soil biota profoundly contribute to plant growth and productivity, and their effects range from positive to negative via respectively mutualistic or antagonistic interactions (Berendsen et al. 2012; van der Putten et al. 2013). Plants, in turn, influence the composition of the soil community around their roots via the excretion of root exudates or sheathing of dead root cells. Plant species can differ greatly in the composition and amount of these deposits, and this can lead to plant species-specific soil communities (Philippot et al. 2013; Shahzad et al. 2015). These specific soil communities can influence the performance of other plants that grow later in the same soil, a process called plant–soil feedback (PSF) (Bever 1994; van der Putten et al. 2013). PSFs can be conspecific, when the plant that grew previously in the soil affects future growth of plants of the same species, or heterospecific, when the plant species that grew previously in the soil affects future growth of other plant species. During the past decade, PSF and its legacy effects have been extensively studied in the context of plant community dynamics, such as environmental change-related range shifts, ecological succession, biological invasion and biodiversity (van der Putten et al. 2013). Recent studies revealed that induced changes in the composition of soil biota by plants could also affect aboveground multitrophic plant–insect interactions (Kostenko et al. 2012; Kos et al. 2015a; Heinen et al. 2018). Moreover, aboveground herbivory in turn can affect the outcome of PSF effects (Heinze and Joshi 2018). The functional group that a plant belongs to may also explain the way in which it influences its soil. Several studies have observed that grasses induce more positive PSF effects than forbs (van de Voorde et al. 2011; Kos et al. 2015b), and that aboveground insect herbivores perform differently on plants growing in forb-conditioned and grass-conditioned soil (Heinen et al. 2018). So far, the mechanistic understanding of how PSFs influence aboveground plant–insect interactions through affecting induced defensive responses in the plant, and how this interacts with aboveground insect herbivory on the plant, remains poorly studied.

To date, a recurring problem in insect–plant research is that most of the knowledge on defense mechanisms, especially defense gene expression, is based on model species (Heidel and Baldwin 2004; de Vos et al. 2006), or on a selected group of economically important plants such as tomato, pepper or maize (Chen et al. 2015). However, some ecologically relevant wild plant species, such as *Jacobaea vulgaris, Plantago lanceolata* and various species in the Brassicaceae family, have been used to study chemical defenses in response to soil biota, which has led to a better understanding of above–belowground ecology (Bezemer et al. 2006a; Soler et al. 2007; Kostenko et al. 2012; Wang et al. 2014, 2015; Kos et al. 2015a). Ribwort Plantain, *P. lanceolata* has a worldwide distribution and has been used as model species addressing plant-mediated above–belowground interactions (e.g., Gange and West 1994; Wurst et al. 2008; Bennett and Bever 2009; Wang et al. 2015). A group of plant secondary defense metabolites that has been well-characterized and well-studied for its ecological role in *P. lanceolata* are iridoid glycosides (IGs). In response to aboveground herbivory and soil biota, such as mycorrhizae or root herbivorous insects, the production of IGs often increases in the plant (Gange and West 1994; Wurst et al. 2008; Bennett and Bever 2009; Schweiger et al. 2014; Wang et al. 2014, 2015). These compounds act as feeding deterrents against generalist herbivores (Puttick and Bowers 1988; Biere et al. 2004; Harvey et al. 2005; Reudler et al. 2011), but can also be used as feeding and oviposition stimulants by specialist herbivores (Bowers and Puttick 1989; Nieminen et al. 2003). Previous studies have examined the effects of addition of single soil organisms on secondary defense responses, but how ‘whole community’ PSF processes influence plant defense has thus far not been studied in detail.

To investigate whether PSF and insect herbivory affect *P. lanceolata* defense responses, we selected four orthologs of genes that are involved in the interactions between plant and biotic agents both above- and belowground. These included a polyphenol oxidase (*Pl* PPO7), a lipoxygenase (*Pl* LOX2-2), and two pathogenesis-related proteins (Pl PR1 and Pl PR2-1). Previous studies have shown that *Pl* LOX2-2 and *Pl* PPO7 are strongly induced in *P. lanceolata* after the application of JA, whereas Pl PR1 and Pl PR2-2 are induced by SA (Figure S1). First, Arabidopsis LOX2 is a key enzyme in the JA biosynthesis pathway induced by (generalist) chewing insect herbivores. LOX2 orthologs are commonly used as markers of JA-mediated defense responses (Chauvin et al. 2013). Second, in several plant species foliar JA-inducible PPOs play a key role in defense against a number of leaf chewing herbivores (Mayer 2006; Bosch et al. 2014). Third, the pathogenesis-related protein PR1 is often used as a marker for SA-mediated disease resistance. It is among the most abundantly produced proteins in plants following infection by biotrophic pathogens (Breen et al. 2017). Finally, PR2 also serves as an SA-marker. Orthologs encode a *ß*-1,3-glucanase that has been proposed to degrade the cell walls of invading fungal pathogens. Possibly PR-proteins like PR-2 have enzymatic activities that generate elicitors of defense responses (van Loon et al. 2006).

In this study, to obtain species-specific conditioned soils, we grew twelve different co-occurring grassland plant species (including the current focal plant *P. lanceolata*) individually in live field collected soil. We then grew *P. lanceolata* in all twelve soils during a feedback phase and exposed a subset of these plants to aboveground herbivory by the chewing insect herbivore *Mamestra brassicae* (Lepidoptera: Noctuidae). We quantified the expression levels of *P. lanceolata* homologues of LOX2, PPO, PR1 and PR2. We also measured concentrations of the defense chemicals aucubin and catalpol (the two major IGs in *P. lanceolata*) in shoots. We address three main questions: (1) Do PSFs of the twelve plant species differ in how they influence the expression of above- and belowground defense-related genes in *P. lanceolata,* and does this interact with the response of the plant to aboveground herbivory? (2) Do PSFs affect chemical defense in *P. lanceolata* leaves? (3) Do PSFs of grasses and forbs differ in how they influence IG levels and defense gene expression in *P. lanceolata* and interact with aboveground herbivory?

## Materials and methods

### Field soil

Field soil was collected from a natural grassland site ‘De Mossel’ (N52°3′, E5°44′, Natuurmonumenten, Ede, The Netherlands). This field has been in use as an experimental field site since 1996 and the soil has been used in numerous plant–soil studies (e.g., Bezemer et al. 2006a, b; Heinen et al. 2018). Live soil was taken from the top 10 cm, the well-rooted layer containing most of the rhizosphere biota. Soil was sieved to remove roots, stones and most macro-invertebrates (sieve mesh Ø1.0 cm).

### Plants and insects

Ribwort Plantain (*P. lanceolata*) was used as a focal species. In previous studies, this species has been shown to be responsive to soil legacies and various biotic players in the soil (Bezemer et al. 2006b; Wurst et al. 2008; Wang et al. 2014, 2015), and its secondary chemistry has been well characterized (Duff et al. 1965; Bowers et al. 1992). RNA transcriptional data (RNAseq) were available for primer design from previous work at the Netherlands Institute of Ecology (A. Biere, unpublished data).

Seeds of *P. lanceolata* were surface-sterilized using 2.5% bleach solution and then rinsed with demineralized water. For germination, seeds were placed on sterile glass beads in a climate cabinet (light regime 16:8, L:D, day temperature 21 °C, night temperature 16 °C). After germination, the seedlings were stored at 4 °C under the same light regime, for later use in experiments. Seeds were obtained from Cruydt-Hoeck (Nijberkoop, The Netherlands).

Eggs of the Cabbage moth, *M. brassicae* were obtained from the Department of Entomology at Wageningen University, The Netherlands. The cabbage moth had been reared for several years on Brussels Sprouts, *Brassica oleracea* var*. gemmifera* cv. Cyrus. The larvae were originally collected from cabbage fields near the university. *M. brassicae* is a generalist chewing herbivore native to the Palearctic. It is known to feed on many species of grasses and forbs, including *P. lanceolata* (Heinen et al. 2018).

### Soil conditioning phase

Twelve common grassland plant species were chosen for soil conditioning, including six forbs: *P. lanceolata* (Plantaginaceae; PL), *Crepis capillaris* (Asteraceae; CC), *Taraxacum officinale* (Asteraceae; TO), *Myosotis arvensis* (Boraginaceae; MA), *Geranium molle* (Geraniaceae; GEM), and *Gnaphalium sylvaticum* (Asteraceae; GS); and six grasses (all Poaceae): *Anthoxanthum odoratum* (AO), *Alopecurus pratensis* (AP), *Holcus lanatus* (HL), *Agrostis capillaris* (AC), *Briza media* (BM), and *Festuca ovina* (FO). Per plant species, five replicate pots were used to condition the soil. Square pots (11 × 11 cm) were filled with 1050 g live field soil topped off with a 0.5 cm layer of fine white sand to prevent oviposition by fungus gnats. In each pot, one seedling was grown for 10 weeks. Plants were kept at 17% soil moisture. After 10 weeks, the plants and their roots were removed from each pot, and the conditioned soil was mixed with sterilized field soil (1:2 conditioned:sterile v/v) to reduce variation in soil nutrient availability, keeping the five replicates separate. Sterile soil was obtained by γ-irradiation (> 25 Kgray, Synergy Health, Ede, The Netherlands), using the live soil that was collected from the field site.

### Feedback phase

New 11 × 11 cm square pots were filled with 1050 g of the mixtures. Two pots were filled with the same soil for each of the replicates in this experiment, one was assigned to the aboveground herbivory treatment and the other one was kept without herbivory (12 conditioned soils, two treatments (herbivore/control), five independent replicates, totaling 120 pots). Each individual pot was planted with a *P. lanceolata* seedling and covered by shade cloth for 3 days. After the seedlings established, the shade cloth was removed. The individual plants were grown for 4 weeks.

### Insect treatment

Plants from both the undamaged control and herbivory treatment were caged using a transparent plastic tube (8 cm Ø; 25 cm high) with a 5-cm mesh covering the top of the cage. Plants allocated to the insect herbivory treatment received one newly hatched L1 *M. brassicae* caterpillar just prior to placing the cage over the plant. The insects were left to feed for 7 days, after which they were removed and the plants were harvested. The removed caterpillars were weighed, and for each plant we measured the absolute leaf area that was consumed by the caterpillar. This was assessed using a visual reference square of 25 mm^2^ (5 × 5 mm) and then estimating the number of times that this visual reference would fit in the total consumed area. The number of squares was multiplied by 25 to get the consumed area per plant in mm^2^.

### Sampling

Immediately after removing the caterpillars, the plants were harvested by clipping the aboveground plant parts with sharp surgical scissors just above soil level. The scissors were cleaned between all clippings with 10% SDS (Biorad, The Netherlands). All leaves of each plant were then folded in aluminum foil and placed in liquid nitrogen before storage in − 80 °C until subsequent sample preparations. Prior to analysis, samples were homogenized per plant in liquid nitrogen and a subsample was taken (fresh) for transcriptome analysis. A second subsample was taken and freeze-dried for use in the chemical analyses.

### Quantitative real-time PCR

Total RNA was isolated and purified from finely ground and homogenized leaf material originating from individual replicate plants with the ISOLATE II RNA Plant Kit (Bioline). Subsequently cDNA was synthesized from RNA (adjusted to 1 µg/µl) using SensiFAST™ cDNA Synthesis Kit (Bioline). To investigate whether PSF and insect herbivory affect *P. lanceolata* defense responses, we selected four genes that are involved in the interactions between plant and biotic agents, including a polyphenol oxidase (*Pl* PPO7), a lipoxygenase (*Pl* LOX2-2), and two pathogenesis-related proteins (*Pl* PR1 and *Pl* PR2-1). *Pl* LOX2-2 and *Pl* PPO7 are induced by JA, involved in signaling of generalist chewing herbivores, whereas Pl PR1 and Pl PR2-2 are induced by SA, involved in signaling of biotrophic pathogens (Figure S1). Gene specific primers were designed using Primer3Plus (http://www.bioinformatics.nl/primer3plus/) and were tested for specificity and efficiency before qPCR experiments. The primer sequences used in this study are listed in Table S1. Quantitative RT-PCR analysis was performed in a CFX96 Touch™ Real-Time PCR Detection System (Bio-Rad). Each reaction was performed in a total volume of 20 µl containing 10 µl SensiFAST SYBR^®^ No-ROX Mix (Bioline), 5 µl cDNA and 1 µl of 400 nM forward and reverse gene specific primer pair. For each reaction, two technical replicates were carried out and average values were used in the analyses. The following PCR program, including a melting curve analysis, was used for all PCR reactions: 3 min 95 °C, followed by 40 cycles of 5 s 95 °C, 10 s 60 °C, and 20 s 72 °C. The normalized expression level of each gene was calculated under the assumption of 100% primer efficiency by means of the 2^−(ΔCt)^ method (formula 7 of Livak and Schmittgen 2001) using the housekeeping gene glyceraldehyde-3-phosphate dehydrogenase (Pl GAPDH) as a reference. The ΔCt values were also used for statistics.

### Iridoid glycosides

To determine iridoid glycoside levels in *P. lanceolata*, plant samples were freeze-dried for 3 days under vacuum (− 55 °C collector temperature; Labconco Free Zone 12 L Freeze Dry System, USA), finely ground and weighed. Twenty-five mg of each sample was extracted overnight in 10 ml, at room temperature in 70% methanol (LichroSolv, VWR) using a horizontal shaker, then filtered and diluted ten times with ultrapure water. The concentrations of the IGs (aucubin and catalpol, Sigma-Aldrich) were analyzed using high-performance liquid chromatography (HPLC, Bioinert 1260 Infinity, Agilent) with electro chemical detection (ECD, Decade elite ECD, Antec). For HPLC quantification, five microliters of filtered extracts and standards was analyzed at 20 °C with a Dionex™ Guard column CarboPac PA1 2 × 50 mm, Main column CarboPac PA1 2 × 250 (Thermo Fisher Scientific). The isocratic mobile phase contained 100% 0.1 M NaOH at a flow rate of 0.25 ml/min, runtime 35 min. Retention time (RT) was 3 and 5 min for aucubin and catalpol, respectively. The standard concentration range was 0.125–2.5 ppm.

### Statistical analyses

Main effects and interactions of ‘soil’ (12 conditioning species) and ‘herbivory’ (herbivory/control) on the relative expression levels (ΔCt) of the four selected *P. lanceolata* genes, as well as the concentrations of IGs (aucubin and catalpol) were analyzed by means of two-way ANOVAs. Post-hoc multiple comparisons were conducted using Tukey–Kramer tests to compare the differences among means if the models were significant.

As the conditioning species consisted of grasses and forbs, we subsequently analyzed the parameters with a general linear mixed model with ‘functional group’ as fixed factor, and ‘soil identity’ (12 conditioning species) as random factor.

The relationship between mean insect growth and consumption per soil treatment and mean levels of catalpol, aucubin, and four defense-related genes was determined using regression analysis.

All analyses were performed in R Studio, R version 3.0.3 (R Development Core Team 2008). Mixed models were performed using the ‘nlme’ package (Pinheiro et al. 2017).

## Results

### Effects on plant biomass

**Fig. 1:**
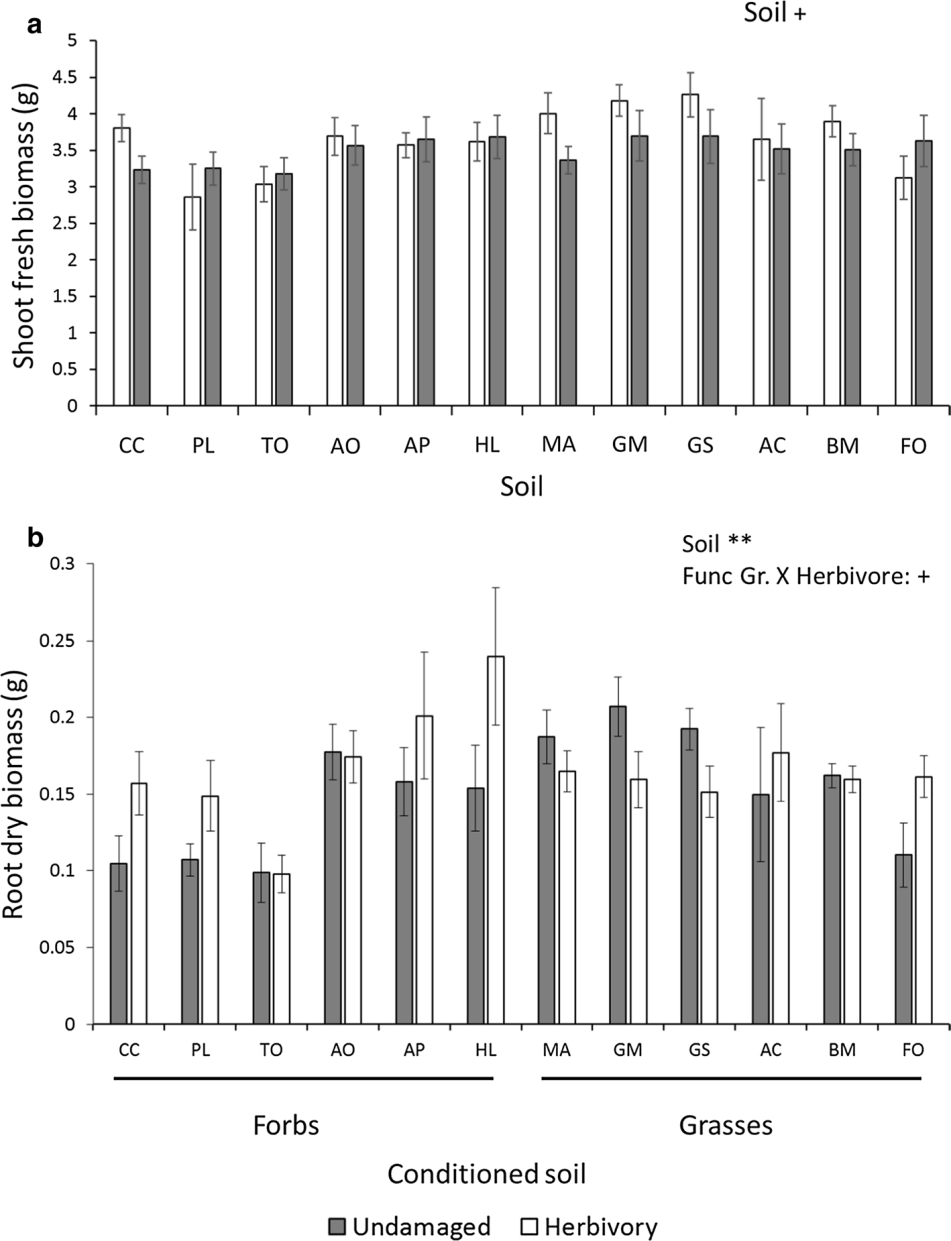
The effects of soil conditioning by twelve common grassland species and herbivory treatments on **a** shoot and **b** root biomass of *Plantago lanceolata.* Grey bars represent undamaged plants and white bars represent plants exposed to herbivory (*Mamestra brassicae*). Error bars represent standard errors. For each treatment combination, *n* = 5. Asterisks represent significant effects; +*p* < 0.07, *p < 0.05, **p < 0.01. Soils were conditioned by either forb or grass species. PL, *Plantago lanceolata*; CC, *Crepis capillaris*; TO, *Taraxacum officinale*; MA, *Myosotis arvensis*; GEM, *Geranium molle*; GS, *Gnaphalium sylvaticum*; AO, *Anthoxanthum odoratum*; AP, *Alopecurus pratensis*; HL, *Holcus lanatus*; AC, *Agrostis capillaris*; BM, *Briza media*; FO, *Festuca ovina*

We found a marginally significant effect of soil on shoot fresh biomass (*F*_11,96_ = 1.8, *p* = 0.065, Fig. 1a). Soil significantly affected *P. lanceolata* belowground dry biomass (*F*_11,96_ = 3.1, *p* = 0.001, Fig. 1b). This effect was driven by the strongly negative effect of *T. officinale*, *C. capillaris* and *P. lanceolata* soils compared to other soils. There also was an almost significant interaction between functional group of the species that conditioned the soil and herbivore treatment (*F*_1,106_ = 3.6, *p* = 0.061). Plants grown on forb-conditioned soils tended to produce more root biomass when they experienced herbivory than control plants, whereas this was not observed for plants grown on grass-conditioned soils (see Fig. 1b).

### Effects on defense related gene expression

**Fig. 2:**
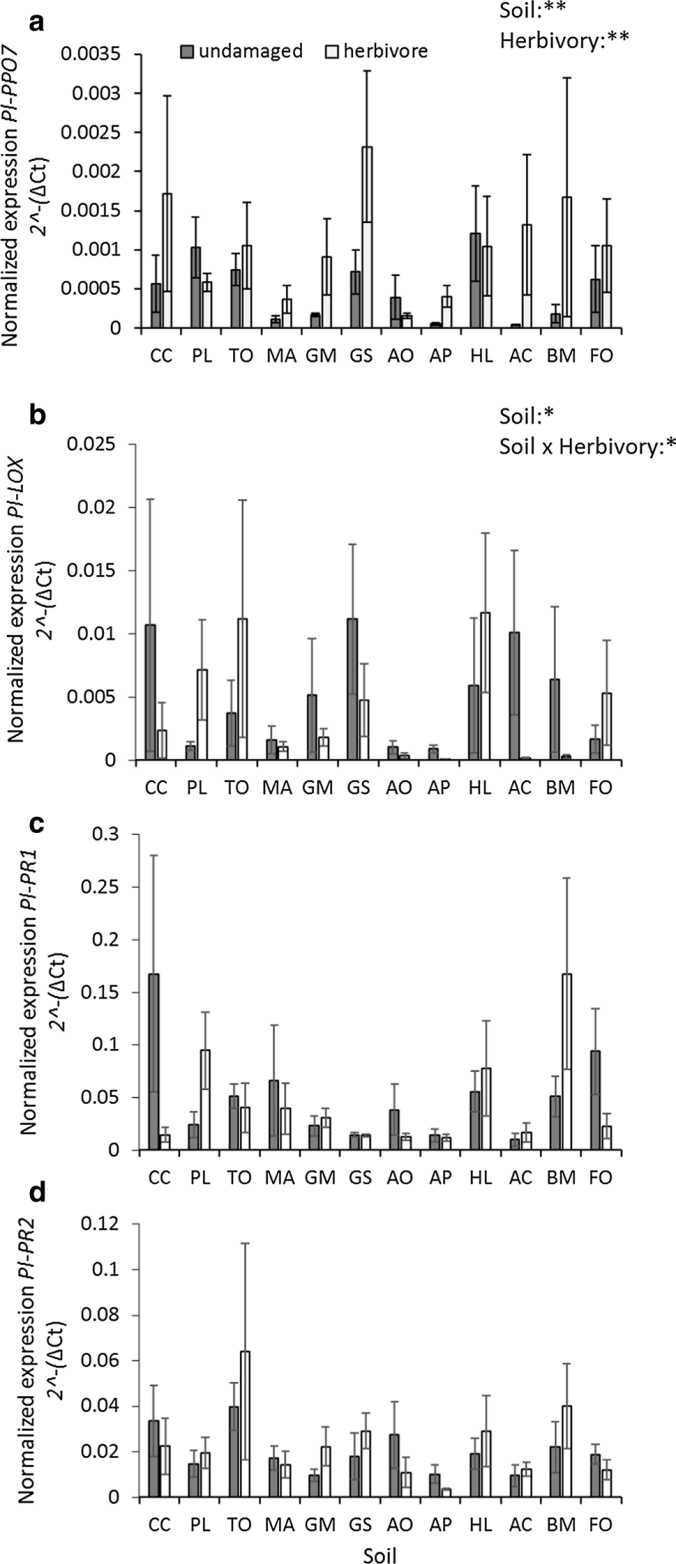
The effects of soil conditioning by twelve common grassland species and herbivory treatments on the relative gene expression levels of four genes in the shoots of *Plantago lanceolata*: *Pl* PPO7 (**a**), *Pl* LOX2-2 (**b**), *Pl* PR1 (**c**) and *Pl* PR2-1 (**d**). Values represent normalized gene expression levels [2^−(ΔCt)^] relative to GAPDH. Grey bars represent undamaged and white bars represent herbivory (*Mamestra brassicae*) treatments. Error bars represent standard errors. For each treatment combination, *n* = 5. Asterisks represent significant effects; *p < 0.05, **p < 0.01. Soils were conditioned by either forb or grass species. PL, *Plantago lanceolata*; CC, *Crepis capillaris*; TO, *Taraxacum officinale*; MA, *Myosotis arvensis*; GEM, *Geranium molle*; GS, *Gnaphalium sylvaticum*; AO, *Anthoxanthum odoratum*; AP, *Alopecurus pratensis*; HL, *Holcus lanatus*; AC, *Agrostis capillaris*; BM, *Briza media*; FO, *Festuca ovina*

Among the four defense-related genes in *P. lanceolata*, the relative expression of *Pl* PPO7 was significantly affected by soil conditioning species and by herbivory (Soil: *F*_11,95_ = 2.87; *p* = 0.003; Herbivory: *F*_1,95_ = 9.73; *p* = 0.002). *Pl* PPO7 expression levels were higher under herbivory treatments, but the levels varied when plants were grown on different soils (Fig. 2a). The expression level was highest when *P. lanceolata* was grown on soils that were previously conditioned by *G. sylvaticum* and lowest on soils conditioned by *M. arvensis*, *A. odoratum* and *A. pratensis.*

A significant interactive effect of herbivory treatment and soil conditioning species was found on the expression of *Pl* LOX2-2 (Herbivory × Soil: *F*_11,96_ = 2.17; *p* = 0.022). The expression was upregulated by herbivory treatment on some soils (i.e., *P. lanceolata, T. officinale, H. lanatus* and *F. ovina*), but downregulated (as compared to caged control plants on the same soils) on soils conditioned by some of the other species (most notably *A. capillaris, B. media, C. capillaris* and *G. sylvaticum*, Fig. 2b).

Expression of *Pl* PR1 and *Pl* PR2-1 was not affected by herbivory treatments, although we found a marginally significant effect of soil on *Pl* PR1 expression (Soil: *F*_11,94_ = 1.87; *p* = 0.053, Fig. 2c and d, Table S2), most likely driven by the high levels found in *P. lanceolata* grown on soils conditioned by *C. capillaris*.

For *Pl* PPO7, the transcript levels were slightly higher in plants that had been grown in forb-conditioned soils compared to those that had been grown in grass-conditioned soils (Functional group: *F*_1,10_ = 4.53; *p* = 0.059).

### Effects on plant chemistry

**Fig. 3:**
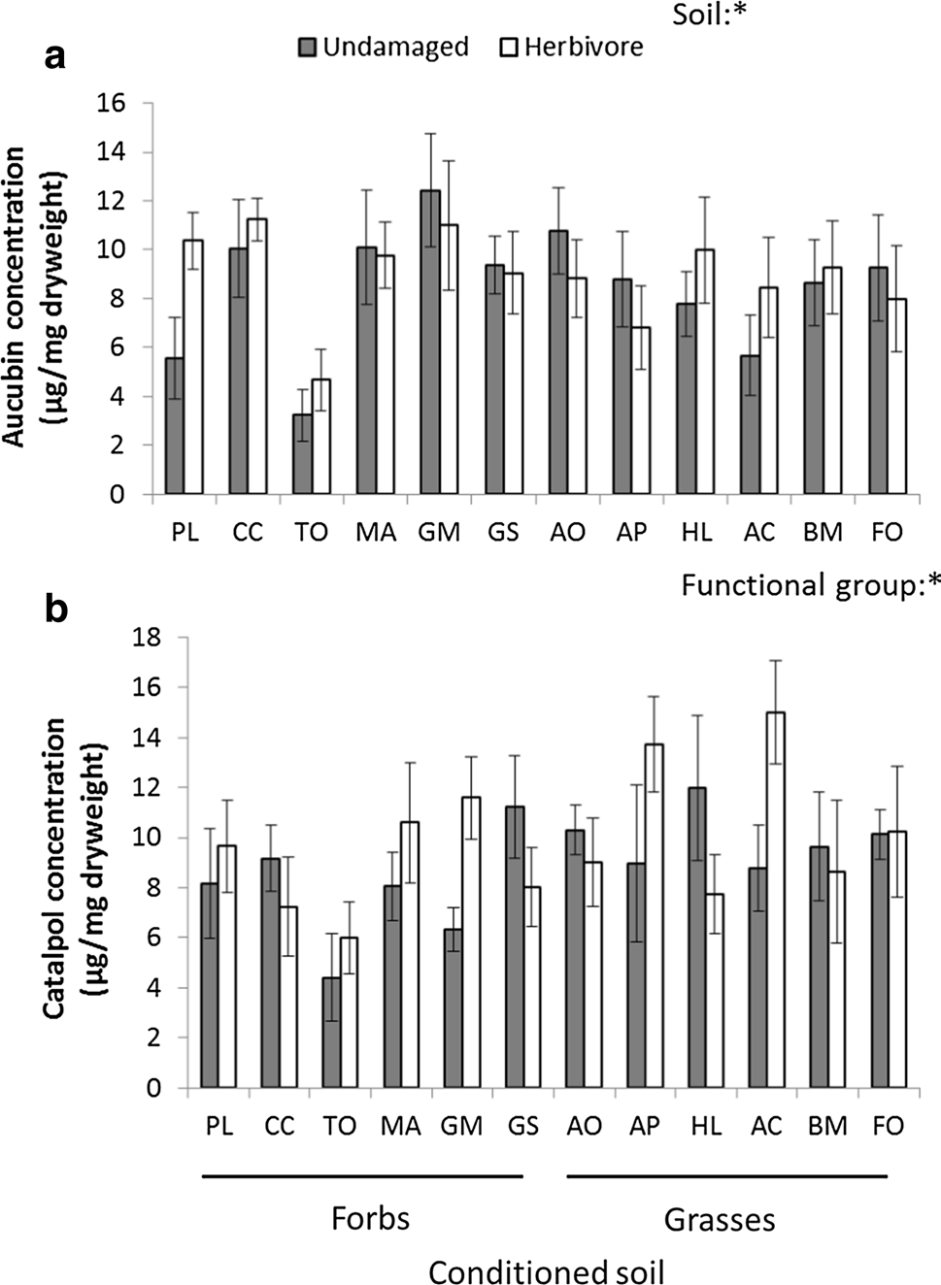
The effects of soil conditioning by twelve common grassland species and herbivory treatment on levels of aucubin (**a**) and catalpol (**b**), in the shoots of *Plantago lanceolata*. Grey bars represent undamaged and white bars represent herbivory (*Mamestra brassicae*) treatments. Error bars represent standard errors. For each treatment combination, *n* = 5. Asterisks represent significant effects; *p < 0.05, **p < 0.01. Soils were conditioned by either forb or grass species. PL, *Plantago lanceolata*; CC, *Crepis capillaris*; TO, *Taraxacum officinale*; MA, *Myosotis arvensis*; GEM, *Geranium molle*; GS, *Gnaphalium sylvaticum*; AO, *Anthoxanthum odoratum*; AP, *Alopecurus pratensis*; HL, *Holcus lanatus*; AC, *Agrostis capillaris*; BM, *Briza media*; FO, *Festuca ovina*

The plant species that conditioned the soil significantly differed in how they affected concentrations of aucubin in shoots of *P. lanceolata* (*F*_11,96_ = 2.40; *p* = 0.011; Fig. 3a). Catalpol was not affected by soil conditioning (Table S2). Aucubin levels of plants grown in soils conditioned by *T. officinale*, were relatively low, whereas levels in soils conditioned by *C. capillaris*, *M. arvensis* and *G. molle* were two to three times higher than those in soils conditioned by *T. officinale* (Fig. 3a). Catalpol levels were significantly higher in *P. lanceolata* plants that were grown on grass-conditioned soils, than those that were grown on forb-conditioned soils (*F*_1,10_ = 5.76; *p* = 0.037, Fig. 3b).

### Effects on caterpillar performance

Species-specific soil legacies did not influence biomass of *M. brassicae* larvae (*F*_11,37_ = 0.57, *p* = 0.84, Figure S2a), nor leaf area consumption by the caterpillars (*F*_11,42_ = 1.27; *p* = 0.28, Figure S2b).

### Correlations between consumption and caterpillar biomass

Caterpillar biomass showed a marginally significant positive correlation with caterpillar consumption (*R*^2^ = 0.33, *p *= 0.052, Supplementary Figure S3).

## Discussion

In this study, we examined how soil legacy effects and aboveground herbivory interact to influence growth and defense responses in the perennial forb *P. lanceolata*. We assessed treatment effects on the transcript levels of four defense-related genes, and measured the production of two secondary defense metabolites, catalpol and aucubin. Our results show that soil conditioning by plants can influence the response of the plant in terms of defense-related gene expression and the production of secondary defense metabolites.

Ribwort plantain, when exposed to *M. brassicae* infestation, showed an up-regulation in transcription of the defense-related gene *Pl* PPO7 that putatively codes for a polyphenol oxidase (PPO). PPOs are known to be induced by herbivory and confer resistance to a broad range of insect herbivores (War et al. 2012). Interestingly, we found that soil conditioning by different plant species also can influence transcript levels of *Pl* PPO7. Moreover, we found an interaction between herbivory and the plant species that conditioned the soil on the overall transcript levels of *Pl* LOX2-2, a gene that is involved in the biosynthesis of JA. *Pl* LOX2-2 was up-regulated by herbivory in some of the conditioned soils, most notably in soils conditioned by *P. lanceolata*, *T. officinale*, *H. lanatus* and *F. ovina*. However, on other soils, herbivory showed no effect on transcript levels of *Pl* LOX2-2, or the gene had a lower expression under the herbivory treatment, compared to control plants (most notably in soils conditioned by *C. capillaris*, *G. sylvaticum*, *A. capillaris* and *B. media*). These results suggest that, at the transcription level, the JA-mediated defensive responses against chewing herbivores may depend on the soil that *P. lanceolata* is growing in. In this study plant material was sampled when the caterpillars had fed on plants for 7 days, thus we were not able to detect the induction of *Pl* LOX2-2 at early stages of herbivory. As lipoxygenase genes are generally considered to respond relatively fast to herbivore damage (Heitz et al. 1997), future studies should follow these induction patterns through a time series.

SA-regulated defense responses are often associated with piercing and sap-sucking insects and with biotrophic and hemibiotrophic phytopathogens (Anand et al. 2008; Pieterse et al. 2014). Soil pathogens are often considered to be important drivers of PSF effects (van der Putten et al. 2013). Therefore, we expected that specific PSFs would affect soil biotic conditions and thereby affect the activation of SA related genes in the plant. In our study, the transcript levels of *Pl* PR2-1, a marker related to the SA signaling pathway, was not strongly affected by the treatments although we found a marginally significant effect of soil conditioning on its homolog marker *Pl* PR1.

Besides harmful pathogens, soils also host microbes that have beneficial relationships with the host plants (Philippot et al. 2013). These beneficial soil microbes, such as mycorrhizae and plant-growth promoting rhizobacteria, have been shown to prime the plant for effective defense responses (Pozo and Azcon-Aguilar 2007; Jung et al. 2012). Soil conditioning likely also influences the compositions of other soil organisms that may alter a plant’s phenotype. Although soil biotic composition was not specifically characterized in this study, in another experiment, performed with the same plant species as we used here, and carried out under similar experimental conditions, plants greatly impacted the structure of soil microbial communities (Heinen et al. 2018).

In the current study, chewing herbivores were used as the inducer of plant defenses. Since chewing herbivores generally invoke the JA pathway rather than the SA pathway (Ali and Agrawal 2012), the absence of an effect of herbivory on the expression of SA-related genes is in line with expectations. Future studies should be conducted to find out whether SA-related gene expression would respond more strongly to soil conditioning when plants are under attack by phloem-feeding herbivores that more commonly induce the SA signaling pathway.

Seeds of Ribwort plantain were not derived from the same genetic background, and plant material used for gene expression analysis was collected from individual *P. lanceolata* replicates. The relative expression values in our study exhibit large variation, indicating strong variability among individual plants in their response to the soils. Most studies on gene expression pool samples from multiple plants, and analyze these pooled samples, which can greatly reduce the variation. We purposely did not pool samples in our study, since individual plants may not respond in the same way and this information cannot be inferred from pooled samples. It may well be that not all individual plants were induced to the same extent. This could be due to differences among individual plants in how they respond to a given set of soil microbes, but also due to differences in the composition of soil organisms among replicate soils. Certain microbes may be present or absent in replicates even though they originated from the same replicate pot with conditioned soil. Nevertheless, even without pooling, our study shows that *P. lanceolata* responded differently to combined soil legacy and herbivory effects with respect to the induction of defense-related genes.

The metabolites aucubin and catalpol have been well-studied in *P. lanceolata* and several studies have shown that both compounds can be induced by herbivory, and by soil organisms (Bowers and Stamp 1993; Marak et al. 2002; Biere et al. 2004; Harvey et al. 2005), such as soil pathogens or arbuscular mycorrhizal fungi (Gange and West 1994; Schweiger et al. 2014; Wang et al. 2015). In this study, *P. lanceolata* secondary defense metabolites were also affected by soil conditioning by twelve different plant species. We only found an effect of soil conditioning species on aucubin levels, which seems to be mainly driven by very low levels of aucubin in *P. lanceolata* growing in soils conditioned by *T. officinale*. In a previous study, *T. officinale* had a negative effect on microbial biomass in the soil (Wardle and Nicholson 1996). As IG levels are often elevated when the plant interacts with microorganisms and nematodes (Wurst et al. 2010), we speculate that differences in IG levels detected may be caused, at least partially, by variation in the activity or community composition of soil organisms. Previous studies have indicated that grasses and forbs differ in their microbial profile in the soil (Kos et al. 2015b; Latz et al. 2015, 2016) and that this can affect aboveground plant–insect interactions (van de Voorde et al. 2011; Kos et al. 2015b; Heinen et al. 2018). In our study, catalpol levels were significantly higher in *P. lanceolata* on soils that were conditioned by grasses, than on those that were conditioned by forbs, regardless of the herbivore treatment. It has also been shown that IGs levels in *P. lanceolata* negatively correlate with nutrient levels available in the soil (Darrow and Bowers 1999; Marak et al. 2003), so a nutritional soil legacy effect cannot be ruled out. In this study, all soils were mixed with two volumes sterilized field soil, which was done to minimize the effect of soil nutritional differences in the feedback phase.

In conclusion, our results shed light on the effect of plant-induced variation in soil biotic and abiotic conditions on defense responses to aboveground herbivory in plants that grow later in these conditioned soils. Until now, mechanisms of how PSF may influence aboveground plant–insect interactions have been highly speculative. Further studies are required, but here we provide evidence that soil legacies can be important drivers of insect–plant interactions—via their influence on plant defense chemistry and the JA-pathway. We showed these effects in a relatively realistic ecological framework, using live soils and natural soil conditioning. Future studies should focus on disentangling the changes in the soil microbiome involved, and manipulating the different classes of soil organisms, such as decomposers, pathogens and beneficial organisms within this framework, to better understand what drives these changes in plant defense.

## Electronic supplementary material

Below is the link to the electronic supplementary material.

Supplementary material 1 (DOCX 126 kb)
