# Supplementary material for: Species-specific plant–soil feedbacks alter herbivore-induced gene expression and defense chemistry in Plantago lanceolata
Source: Oecologia. 2018 Aug 14;188(3):801–11. doi: 10.1007/s00442-018-4245-9 (PMC6208702; doi:10.1007/s00442-018-4245-9)

**Electronic Supplemental Material**

| Table S1. Specific primer sequences used for quantitative RT-PCR analyses. | | |
| --- | --- | --- |
| Gene name | Forward primer | Reverse primer |
| *Pl* GAPDH | AGCAAGCTTCCCACCTTCTC | TGGGAATGTCACCCTTTCCG |
| *Pl* PPO7 | TTTCCTGGAATCGGAGTTTG | GGTTGCGCGTCTATCTTAGC |
| *Pl* LOX2-2 | CCTCAGTCCTCTCCAAACTCA | GGTTGGGAGCAAAGGCTTAT |
| *Pl* PR1 | CGCAAGGAACTATGCACAAA | ACTCTCCTCCAACGCAAGAA |
| *Pl* PR2-1 | CCCGGCTTATAGTTTCCACA | CTCCAGAGCCGGTGTAAGAG |

| Table S2: Statistical results of the effect of herbivory, soil legacy and functional group of conditioning plant species on induction of defense-related genes in *Plantago lanceolata*. Shown are degrees of freedom, F-value and P-value of a two-way ANOVA with soil (conditioning species) and herbivory treatment (herbivory/control) as factors and the output of a general linear mixed model with functional group of the conditioning species (grass/forb) and herbivory treatment (herbivory/control) as fixed factors and soil as random factor. | | | | | |
| --- | --- | --- | --- | --- | --- |
| Plantago gene | Model | Model factors | Degrees of freedom | F-value | P-value |
| ***Pl* PPO7_a_** |  |  |  |  |  |
|  | Two-way | herbivory | 1, 95 | 9.73 | **0.002** |
|  | ANOVA | soil | 11, 95 | 2.87 | **0.003** |
|  |  | herbivory x soil | 11, 95 | 1.29 | 0.241 |
|  |  |  |  |  |  |
|  | GLMM | herbivory | 1, 105 | 9.43 | **0.003** |
|  |  | grass-forb | 1, 10 | 4.53 | *0.059* |
|  |  | herbivory x grass-forb | 1, 105 | 0.53 | 0.468 |
| ***Pl* LOX2-2_a_** |  |  |  |  |  |
|  | Two-way | herbivory | 1, 96 | 1.54 | 0.217 |
|  | ANOVA | soil | 11, 96 | 1.91 | **0.048** |
|  |  | herbivory x soil | 11, 96 | 2.17 | **0.022** |
|  |  |  |  |  |  |
|  | GLMM | herbivory | 1, 106 | 1.38 | 0.242 |
|  |  | grass-forb | 1, 10 | 3.82 | *0.079* |
|  |  | herbivory x grass-forb | 1, 106 | 3.16 | *0.079* |
| ***Pl* PR1_a_** |  |  |  |  |  |
|  | Two-way | herbivory | 1, 94 | 0.07 | 0.797 |
|  | ANOVA | soil | 11, 94 | 1.87 | *0.053* |
|  |  | herbivory x soil | 11, 94 | 1.30 | 0.235 |
|  |  |  |  |  |  |
|  | GLMM | herbivory | 1, 104 | 0.06 | 0.811 |
|  |  | grass-forb | 1, 10 | 0.36 | 0.564 |
|  |  | herbivory x grass-forb | 1, 104 | 0.28 | 0.600 |
| ***Pl* PR2-1_a_** |  |  |  |  |  |
|  | Two-way | herbivory | 1, 95 | 0.05 | 0.821 |
|  | ANOVA | soil | 11, 95 | 1.61 | 0.108 |
|  |  | herbivory x soil | 11, 95 | 0.91 | 0.536 |
|  |  |  |  |  |  |
|  | GLMM | herbivory | 1, 105 | 0.05 | 0.822 |
|  |  | grass-forb | 1, 10 | 1.82 | 0.207 |
|  |  | herbivory x grass-forb | 1, 105 | 0.02 | 0.881 |
| a) Values were log-transformed prior to statistical analysis. | | | | | |

| Table S3: Statistical results of the effect of herbivory, soil legacy and functional group of conditioning plant species on production of iridoid glycosides (aucubin and catalpol) in *Plantago lanceolata*. Shown are degrees of freedom, F-value and P-value of a two-way ANOVA with soil (conditioning species) and herbivory treatment (herbivory/control) as factors and the output of a general linear mixed model with functional group of the conditioning species (grass/forb) and herbivory treatment (herbivory/control) as fixed factors and soil as random factor. | | | | | |
| --- | --- | --- | --- | --- | --- |
| IGs | Model | Model factors | Degrees of freedom | F-value | P-value |
| **aucubin** |  |  |  |  |  |
|  | Two-way | herbivory | 1, 96 | 0.43 | 0.513 |
|  | ANOVA | soil | 11, 96 | 2.40 | **0.011** |
|  |  | herbivory x soil | 11, 96 | 0.68 | 0.752 |
|  |  |  |  |  |  |
|  | GLMM | herbivory | 1, 106 | 0.44 | 0.511 |
|  |  | grass-forb | 1, 10 | 0.12 | 0.736 |
|  |  | herbivory x grass-forb | 1, 106 | 0.32 | 0.576 |
|  |  |  |  |  |  |
| **catalpol** |  |  |  |  |  |
|  | Two-way | herbivory | 1, 96 | 1.14 | 0.288 |
|  | ANOVA | soil | 11, 96 | 1.44 | 0.170 |
|  |  | herbivory x soil | 11, 96 | 1.49 | 0.148 |
|  |  |  |  |  |  |
|  | GLMM | herbivory | 1, 106 | 1.08 | 0.300 |
|  |  | grass-forb | 1, 10 | 5.76 | **0.037** |
|  |  | herbivory x grass-forb | 1, 106 | 0.01 | 0.904 |

Figure S1: Relative gene expression of *Plantago lanceolata* homologues of PPO, LOX2, PR-1, and PR-2 used in the experiment. Data from an unpublished RNA-seq experiment (Illumina Hi-seq100 paired end) in which the fourth-youngest fully expanded leaves of seven-week old plants were induced with 250 uL of jasmonic acid (10 mM; J), salicylic acid (5 mM; S), or mock treatment with acid water (C). Values are mean ± s.e. fold changes in expression of J and S plants compared to the control C, based on n=6 biological replicates of 9 pooled plants each (A. Biere, unpublished data). Stars indicate significant differences from the control (* P<0.05; ** P<0.01; *** P<0.001). Closest homologues in *Arabiopsis thaliana*: *Pl* PPO-7 (576 identity): no homology; closest homologue *Sesamum indicum* polyphenol oxidase 1, chloroplastic-like (66% identity); *Pl* LOX2-2 (907 nucleotides) lipoxygenase *At*LOX2 (55% identity); *Pl* PR1 (161 nucleotides): basic pathogenesis-related protein 1 (59% similarity); *Pl* PR2-1 (341 nucleotides): beta-1,3 glucanase 1, PR-2 (53% identity).

**
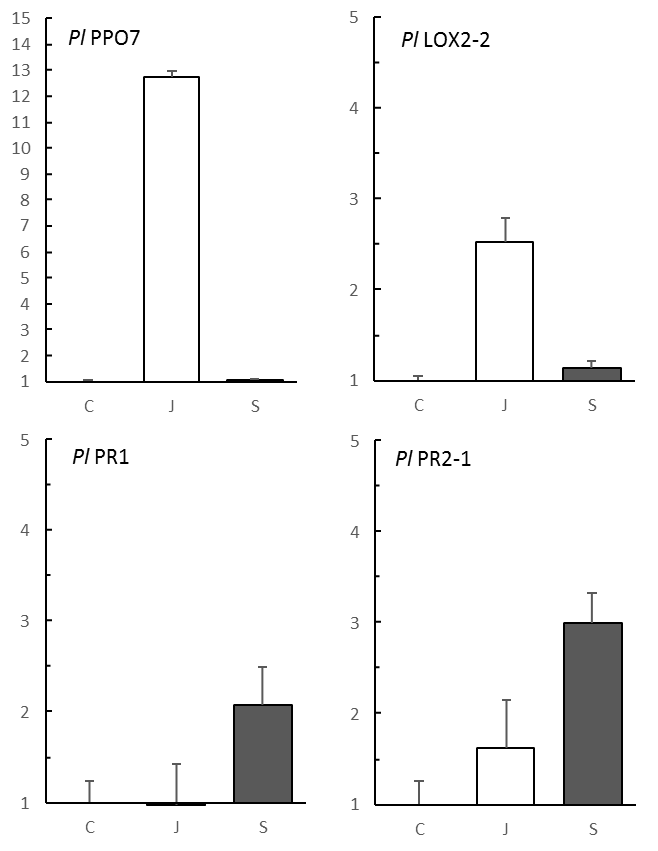
**

Figure S2: The effects of soil conditioning by twelve common grassland species on biomass of *Mamestra brassicae* (A), and herbivore consumption (B), feeding on *Plantago lanceolata*. Error bars represent standard errors. For each treatment combination, n=5. Soils were conditioned by either forb or grass species. Abbreviations: PL = *Plantago lanceolata*, CC = *Crepis capillaris*, TO = *Taraxacum officinale*, MA = *Myosotis arvensis*, GEM = *Geranium molle*, GS = *Gnaphalium sylvaticum*, AO = *Anthoxanthum odoratum*, AP = *Alopecurus pratensis*, HL = *Holcus lanatus*, AC = *Agrostis capillaris*, BM = *Briza media*, FO = *Festuca ovina*.


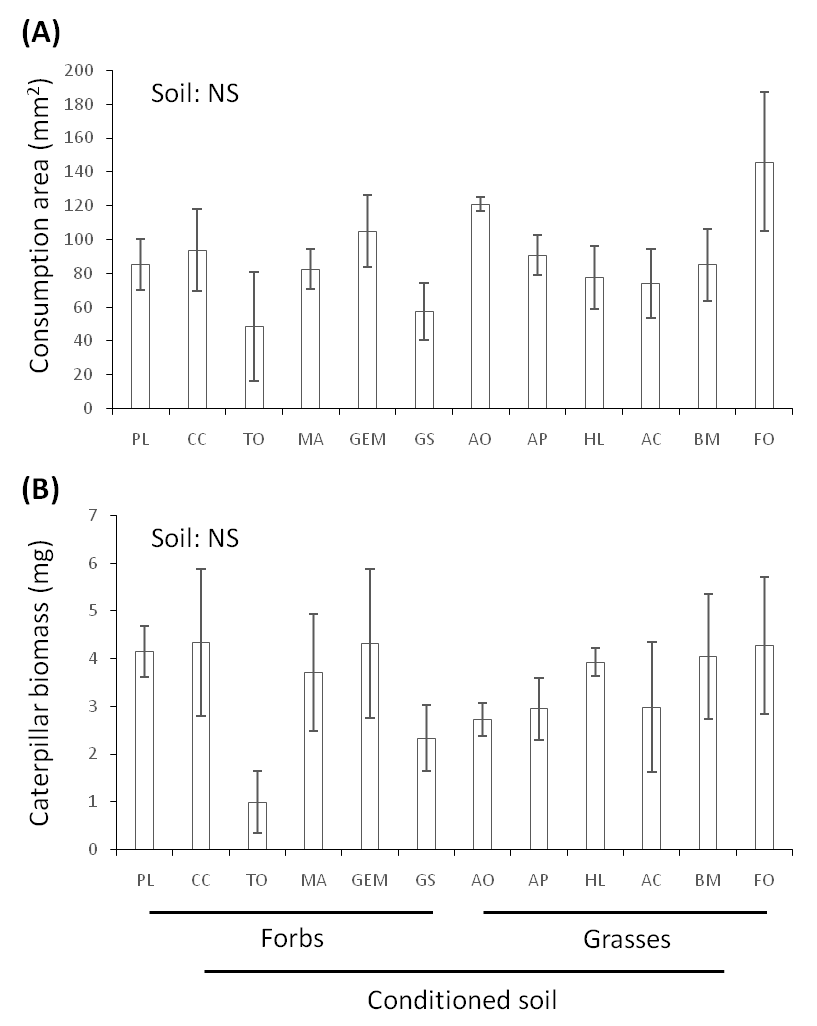


Figure S3: Correlation between mean caterpillar biomass and consumption area in the shoot of *Plantago lanceolata*. Each data point represents the average caterpillar biomass and consumption area for one conditioned soil. For each average, n=5.


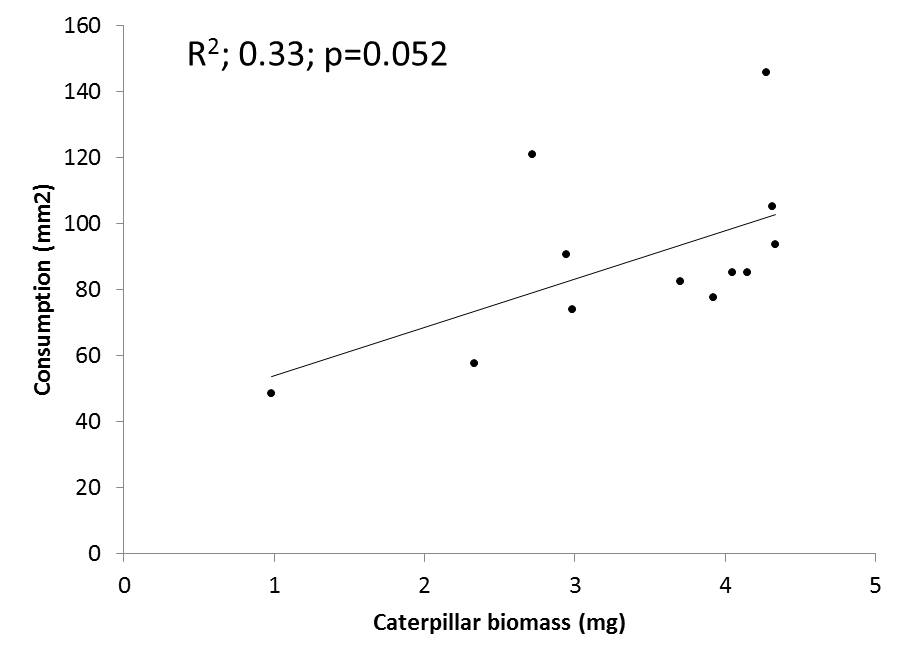

Supplement: Supplementary file 1 — Supplementary material 1 (DOCX 126 kb) [file 442_2018_4245_MOESM1_ESM.docx]
